# Population and Individual-Level Double Burden of Malnutrition Among Adolescents in Two Emerging Cities in Northern and Southern Nigeria: A Comparative Cross-Sectional Study

**DOI:** 10.5334/aogh.3093

**Published:** 2020-12-14

**Authors:** Oghenebrume Wariri, Kingsley Irelosen Akhimienho, Jacob Albin Korem Alhassan, Iliya Jalo, Iso Precious Oloyede, Eno Etim Nyong, Fidelia Bode-Thomas

**Affiliations:** 1 MRC Unit the Gambia at the London School of Hygiene and Tropical Medicine, Fajara, GM; 2 African Population and Health Policy Initiative, Gombe, NG; 3 Aberdeen Centre for Health Data Science (ACHDS), Institute of Applied Health Sciences, University of Aberdeen, UK; 4 Department of Paediatrics, University of Uyo Teaching Hospital, Akwa-Ibom State, NG; 5 Department of Community Health and Epidemiology, College of Medicine, University of Saskatchewan, CA; 6 Department of Paediatrics, Federal Teaching Hospital Gombe, NG; 7 Department of Paediatrics, College of Medical Sciences, Gombe State University, Gombe, NG; 8 Paediatric Cardiology Unit, Department of Paediatrics, Jos University Teaching Hospital (JUTH), Jos, NG

## Abstract

**Background:**

Over the past three decades, double burden of malnutrition (DBM), a situation where high levels of undernutrition (stunting, thinness, or micronutrient deficiency) coexist with overnutrition (overweight and obesity), continues to rise in sub-Saharan Africa. Compared to other countries in the region, the evidence on DBM is limited in Nigeria.

**Objective:**

This paper aimed to determine the comparative prevalence of population-level and individual-level DBM among adolescents in two emerging cities in northern and southern Nigeria.

**Methods:**

This was a comparative cross-sectional study among apparently healthy secondary school adolescents aged 10–18 years in Gombe (northern Nigeria) and Uyo (southern Nigeria) between January 2015 and June 2017. A multistage random sampling technique was implemented to recruit adolescents from 24 secondary schools in both cities. Measures of general obesity (body mass index) and stature (height-for-age) were classified and Z-scores generated using the WHO *AnthroPlus* software, which is based on the WHO 2006 growth reference. Population-level DBM was defined as the occurrence of thinness and overweight/obesity within the population. Individual-level DBM was defined as the proportion of individuals who were concurrently stunted and had truncal obesity or stunted and were overweight/obese.

**Findings:**

Overall, at the population-level in both settings, 6.8% of adolescents had thinness, while 12.4% were overweight/obese signifying a high burden of population-level DBM. Comparatively, the population-level DBM was higher in Gombe compared to Uyo (thinness: 11.98% vs 5.3% and overweight/obesity: 16.08% vs 11.27% in Gombe vs Uyo respectively). Overall, at the individual level, 6.42% of stunted adolescents had coexisting truncal obesity, while 8.02% were stunted and had coexisting general overweight/obesity. Like the trend with population-level DBM, individual-level DBM was higher in Gombe (northern Nigeria) compared to Uyo (southern Nigeria).

**Conclusion:**

High levels of population-level and individual-level DBM exist in Gombe and Uyo. However, the level of DBM (under- and over-nutrition) is higher in Gombe located in northern Nigeria compared to Uyo in southern Nigeria.

## Background

Globally, more than 800 million people are affected by chronic undernutrition (stunting and wasting), with sub-Saharan Africa (SSA) disproportionately represented [1]. In 2017 alone, the prevalence of stunting among children in SSA was 30.3% [2]. Meanwhile, over the last two decades, the number of children with overnutrition in SSA rose steeply, from 5.4 million in 1990 to 10.3 million in 2015 [3], with a prevalence of overweight/obesity of 6.8% among preschool children in 2010 [4]. Instead of the two distinct silos of under-nutrition or over-nutrition thought to be restricted to countries of the global south or global north respectively, a double burden of malnutrition (DBM) has emerged globally [5,6,7], with an increasing prevalence in many low- and middle-income (LMICs) [8,9]. DBM is defined as a situation where high levels of undernutrition (underweight, childhood stunting, and wasting) coexist with overnutrition (overweight and obesity) at the individual, household/family, or population-level [10]. The number of countries in SSA with an increasing burden of the DBM continue to rise [11].

The underlying causes of the shift towards DBM in SSA vary across subregions and across national borders. The nutritional epidemiological shift is thought to be shaped by drivers such as rapidly changing diets manifested through increasing consumption of cheap, highly processed energy-dense foods with low nutritional value, and high fats in societies with chronic background undernutrition (especially stunting) [12]. Rapid economic transitions, urbanisation, cultural expectations in body size, and reduced physical activity are also key drivers of the DBM in SSA [9,11,12]. The drivers of the DBM, thus, originate predominantly outside the health system but have manifold consequences for health systems in SSA, already struggling to cope with threats from infectious diseases, maternal and child health issues. DBM poses several long-term complications, especially if people developed DBM during childhood and adolescence, as it increases the risk of diverse chronic non-communicable diseases (NCDs) [12].

Evidence of the DBM among children in SSA is emerging, though still limited [13]. Most of the research have focused on children under five years old (U5) and women of reproductive age (15–49 years) with limited data specifically on adolescents [14]. Furthermore, ‘population-level’ and ‘household-level’ DBM have received considerable research attention [9,15,16]. Population-level DBM refers to a high prevalence of under-nutrition and the over-nutrition within specific populations (communities, countries), while household-level DBM is, in its simplest form, the coexistence of an overweight/obese mother and a stunted child at the same time in a household [10]. There remains a paucity of research on ‘individual-level’ (i.e. the coexistence of undernutrition such as stunting and overweight/obesity in one individual) DBM in SSA settings.

Compared to other countries in SSA, there is limited literature on the burden of DBM in Nigeria [17,18]. The existing literature on DBM in Nigeria is limited to household/population-level DBM or focussed on women of reproductive age, and U5 children [17,18]. To the authors’ knowledge, there are no published data on the burden of DBM in northern Nigeria. Furthermore, there are no studies comparing the burden of DBM between northern and southern Nigeria. Comparisons between northern and southern Nigeria could provide critical evidence to illuminate intra-country variations in the epidemiological and nutritional transitions as different locations might be at different stages, due, for example, to varying economic realities prevalent in northern compared to southern Nigeria. To contribute to bridging the evidence gap, this paper aimed to; 1) determine the comparative prevalence of population-level DBM and 2) the comparative prevalence of individual-level DBM among adolescents in two emerging cities in northern and southern Nigeria. The comparison is based on one measure of general adiposity – the body mass index (BMI), a measure of truncal adiposity – the waist-to-height-ratio (WHtR), and height-for-age which measures stunting, a form of chronic under-nutrition.

## Methods

### Study Design

This was a consecutive cross-sectional study conducted among apparently healthy secondary school adolescents in two rapidly developing Nigerian cities and compared their findings. First, in Gombe (northern Nigeria) and subsequently in Uyo (southern Nigeria) between January 2015 and June 2017. The design, conduct, and reporting of findings followed the Strengthening the Reporting of Observational Studies in Epidemiology (STROBE) checklist for cross-sectional studies [19].

### Study Setting and Population

Gombe is the capital city of Gombe State in northern Nigeria, with a population density of 148 per km^2^ [20]. Uyo is the capital city of Akwa-Ibom State in southern Nigeria and has a population density of 653.2 per km^2^, which is more than four times that of Gombe State [21]. In 2015, Gombe City had an estimated population of 417,000 people with an annual population growth rate of 4.05% [22]. In the same year, Uyo city had an estimated population of 847,500 people (double that of Gombe city) and an annual population growth rate of 6.02% [23]. Gombe city has 49 secondary schools (20 public and 29 private) compared to 145 secondary schools (14 public and 131 private) in Uyo city. The overwhelming majority of health facilities in Gombe and Uyo City are primary and secondary health centres which provide basic preventive and curative care and funded by the sub-national government or private for-profit organisations. Both cities have one tertiary-level teaching hospital that provide specialist care, including adolescent health services.

### Sampling

Due to varying epidemiologic, geographic, and economic dynamics prevalent in the study settings, the minimum sample sizes were estimated separately for Gombe and Uyo. In Gombe, the sample size was computed based on a prevalence of overweight/obesity of 2.82% from a previous study in Kano, northern Nigeria which is geographically and economically similar to Gombe [24]. In estimating the 377 adolescents studied in Gombe, a power of 80%, degree of precision of 2% [25], a 95% confidence interval, and a 10% parental refusal rate or incomplete data was assumed. In Uyo, the sample size was estimated based on a prevalence of obesity of 4.2% reported in a previous study in Ile-Ife, southern Nigeria which is economically similar to Uyo [26]. In estimating the 1,740 adolescents studied in Uyo, a power of 80%, degree of precision of 2.0% [25], a 95% confidence interval, and a 20% parental refusal rate or incomplete data was assumed.

A multistage random sampling technique was implemented in this study to recruit 10–18 years old adolescents, from a total of 24 secondary schools; i.e. 12 each in Gombe and Uyo respectively. The sampling technique employed in this study has been described in details in a previously published sub-study based on data from the larger study [27]. In brief, schools included in the study were randomly selected from a government-approved list of secondary schools, ensuring private and public schools were appropriately represented. The number of pupils recruited from the included schools, and subsequently from each grade, was determined by the proportionate sampling method.

### Inclusion and Exclusion Criteria

Apparently healthy secondary school adolescents aged 10–18 years who assented to the study after their parents had consented to the study were included. Participants with any chronic illness ascertained based on participant volunteered information, available school records, or evidence from physical examination were excluded. Participants with haematuria and glucosuria on urinalysis, those who actively consumed alcohol or cigarette within three months before the study, and those who were on medications such as steroids and diuretics were also excluded.

### Data Collection and Anthropometry

Participant and parental sociodemographic parameters including age, sex, drug history, ethnicity, parental education, and occupation were collected using pre-tested researcher administered questionnaires. Anthropometric measurements were conducted in the school premises by the researchers and trained assistants using standardized and validated procedures [28]. Participants removed all heavy outer clothing and accessories including sweaters, shoes, wristwatches, belts and emptied their pockets before anthropometric measurements were done. Using a digital scale (Seca® 877 Class III), body weight was measured to the nearest 0.1 kg. Height was measured to the nearest 0.1 cm using a collapsible stadiometer (Seca® Leicester portable height measure), while waist circumference was measured with a non-stretch tape rule placed horizontally, midway between the lower border of the 10^th^ rib and iliac crest. All measurements were conducted in duplicate and the mean was considered. BMI-for-age z-scores (BAZ) and height-for-age z-scores (HAZ) were generated using the WHO *AnthroPlus* software, which is based on the WHO 2006 growth reference charts for 5–19 years old children [29].

### Derived Variables and Definition of Outcome Variables

BMI was calculated by dividing body weight (in kilograms) by height (in metre square), while WHtR was derived by dividing waist circumference in centimetres by height in centimetres. Nutritional status of participants was determined using the WHO 2006 growth reference for children 5–19 years. BAZ of < –3SD was defined as severe thinness, < –2SD was thinness, ≥ –2 to ≤ +1SD was normal, > +1SD was overweight, and > +2SD was obese [30]. Severely stunted was defined as HAZ < –3SD, stunted was < –2SD, and normal stature was ≥ –2 to ≤ +2SD [30]. WHtR of < 0.5 was classified as normal and ≥ 0.5 was truncal obesity [31].

### Double Burden Definitions

To determine population-level and individual-level DBM, BAZ categories were collapsed into three; ‘thinness’ (combined severe thinness and thinness), ‘normal’, and ‘overweight/obesity’ (combined overweight and obesity). Also, HAZ categories were collapsed into two; ‘stunted’ (combined severely stunted and stunted) and ‘normal’. Population-level DBM was defined as the occurrence of ‘thinness’ and ‘overweight/obesity’ (based on BAZ categorization) within the population in Gombe and Uyo. Individual-level DBM was defined and computed as the proportion of participants (individuals) who were concurrently ‘stunted’ (HAZ < –2SD) and ‘Obese’ [i.e. truncal obesity (WHtR ≥ 0.5) or general overweight/obesity (BAZ > +1SD)].

### Statistical Analysis

All data generated were processed and analysed using STATA 16 [32]. Age was sub-categorized into three; early adolescence (10–13 years), middle adolescence (14–16 years), and late adolescence (17–19 years) [33]. Family socioeconomic status (SES) was computed as a composite score ranging from one (wealthiest) to five (poorest) based on the level of maternal education and paternal occupation using the method described by Olusanya et al. [34]. Categorical variables were reported as counts and percentages. Continuous variables were reported as mean and 95% confidence intervals (CI). For group comparisons, cross-tabulations with the outcome were performed using the χ^2^ statistic for categorical variables and independent sample t-test for continuous variables. Statistical significance was defined as alpha < 0.05 (two-sided).

## Results

### General Population Characteristics

A total of 2,100 children and adolescents were included in this analysis, made up of 1,733 in Uyo (southern Nigeria) and 367 from Gombe (northern Nigeria). Seven of the sampled adolescents in Uyo and 10 in Gombe were excluded from the analysis due to incomplete data. The majority (50.29%) were in the middle adolescence category (i.e. aged 14–16 years) and mean age of 14.38 (95% CI = 14.29, 14.47). The sample included more females (55.86%), more adolescents who identified as Christians (89.38%), and more adolescents from monogamous households (82.43%). About 2% of participants belonged to the poorest socio-economic quintile and 25.33% belonged to the wealthiest quintile. By ethnicity, the adolescents in Uyo were predominantly Ibibios (81.36%) while those in Gombe were mainly Fulani (33.24%) (Table 1).

**Table 1:** Comparison of participants by various sociodemographic characteristics among 2,100 adolescents in Gombe City and Uyo City in northern and southern Nigeria respectively.

Variable	Uyo City	Gombe City	Total (Overall)	P Value

	1733 (%)	367 (%)	2,100 (%)	

**Age group**				
Early (10–13)	595 (34.33)	89 (24.25)	684 (32.57)	
Middle (14–16)	876 (50.55)	180 (49.05)	1,056 (50.29)	
Late (17–18)	262 (15.12)	98 (26.70)	360 (17.14)	0.000
**Sex**				
Male	736 (42.47)	191 (52.04)	927 (44.14)	
Female	997 (57.53)	176 (47.96)	1,173 (55.86)	0.001
**Religion**				
Christianity	1,724 (99.48)	153 (41.69)	1,877 (89.38)	
Islam	9 (0.52)	214 (58.31)	223 (10.62)	0.000
**Ethnicity**				
Ibibio	1,410 (81.36)	0 (0.00)	1,410 (67.14)	
Igbo	109 (6.29)	3 (0.82)	112 (5.33)	
Annang	60 (3.46)	0 (0.00)	60 (2.86)	
Oro	44 (2.54)	0 (0.00)	44 (2.10)	
Efik	46 (2.65)	0 (0.00)	46 (2.19)	
Hausa	12 (0.69)	85 (23.16)	97 (4.62)	
Fulani	0 (0.00)	122 (33.24)	122 (5.81)	
Tangale	0 (0.00)	69 (18.80)	69 (3.29)	
Waja	0 (0.00)	20 (5.45)	20 (0.95)	
Yoruba	19 (1.10)	15 (4.09)	34 (1.62)	
Tera	0 (0.00)	13 (3.54)	13 (0.62)	
Others	33 (1.90)	40 (10.90)	73 (3.48)	0.000
**Family Setting**				
Monogamous	1,488 (85.86)	243 (66.21)	1,731 (82.43)	
Polygamous	245 (14.14)	124 (33.79)	369 (17.57)	0.000
**Family Socioeconomic status**				
1 (Wealthiest)	493 (28.45)	39 (10.63)	532 (25.33)	
2	536 (30.93)	70 (19.07)	606 (28.86)	
3	465 (26.83)	152 (41.42)	617 (29.38)	
4	221 (12.75)	83 (22.62)	304 (14.48)	
5 (Poorest)	18 (1.04)	23 (6.27)	41 (1.95)	0.000
Age (years), Mean (95%CI)	14.27 (14.17, 14.28)	14.92 (14.71, 15.11)	14.38 (14.29, 14.47)	0.000
Weight (kg), Mean (95%CI)	48.79 (48.32, 49.31)	49.65 (48.40, 50.90)	48.94 (48.49, 49.39)	0.156
Height (cm), Mean (95%CI)	157.72 (157.27, 158.16)	157.70 (156.70, 158.71)	157.72 (157.31, 158.12)	0.967
Waist (cm), Mean (95%CI)	69.47 (68.69, 70.25)	68.73 (67.71, 69.72)	69.34 (68.68, 70.01)	0.404
BMI, Mean (95%CI)	19.48 (19.34, 19.63)	19.85 (19.42, 20.27)	19.55 (19.41, 19.69)	0.053
WHtR, Mean (95%CI)	0.44 (0.44, 0.45)	0.44 (0.43, 0.44)	0.44 (0.44, 0.44)	0.423
BMI for age Z score, Mean (95%CI)	–0.24 (–0.30, –0.19)	–0.37 (–0.52, –0.24)	–0.27 (–0.32, –0.21)	0.049
Height for Age Z scores, Mean (95%CI)	–0.46 (–0.52, –0.41)	–0.37 (–0.98, –0.75)	–0.53 (–0.58, –0.48)	0.000

+ All confidence intervals and P Values from mean age to mean height for age z scores were generated after performing an independent sample t test comparing north and south.

### Anthropometric Measures

Basic measures of anthropometry showed northern and southern children to be similar. The average weight of participants was 48.94kg with no statistically significant difference between northern and southern participants (p = 0.156). The average height which was 157.72 cm showed no statistically significant differences as well (p = 0.967), although southern adolescents included in the analyses had marginally wider waist circumferences with an average of 69.47 ± 0.78 compared to their northern counterparts 69.34 ± 0.34 (p = 0.404). Overall, the average BMI of 19.55 showed no statistically significant difference between adolescents in the north and their counterparts in the south (p = 0.53). Average WHtR was 0.44 with no statistically significant difference between northern and southern Nigerian adolescents (p = 0.423). Mean BAZ averaged –0.27 ± 0.05 (p = 0.049) and HAZ averaged –0.53 ± 0.08 (p = 0.000) with statistically significant differences between northern and southern adolescents (Table 1).

### Population-level DBM

**Figure 1:**
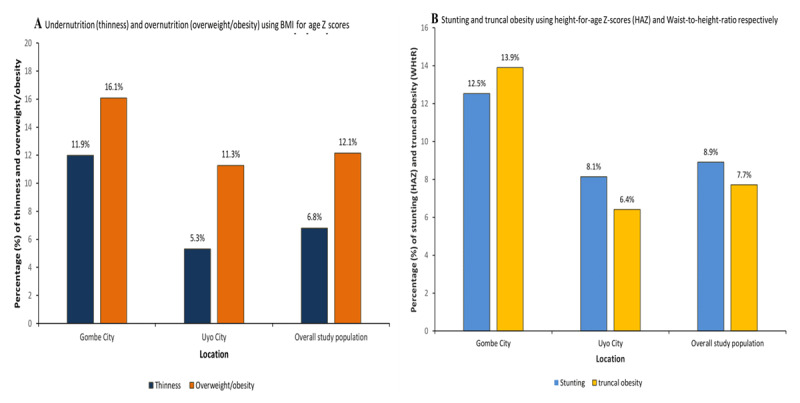
Prevalence rate of population-level thinness, overweight/obesity, stunting, and truncal obesity among adolescents in Gombe, Uyo and the overall study population. *Note*: Thinness = BAZ < –2SD, overweight/obesity = BAZ > 1SD, stunting = HAZ < –2SD, and truncal obesity = WHtR ≥ 0.5. BAZ (BMI-for-age Z-scores), HAZ (Height-for-age Z-scores) and WHtR (Weight-to-Height-Ratio).

**Figure 2:**
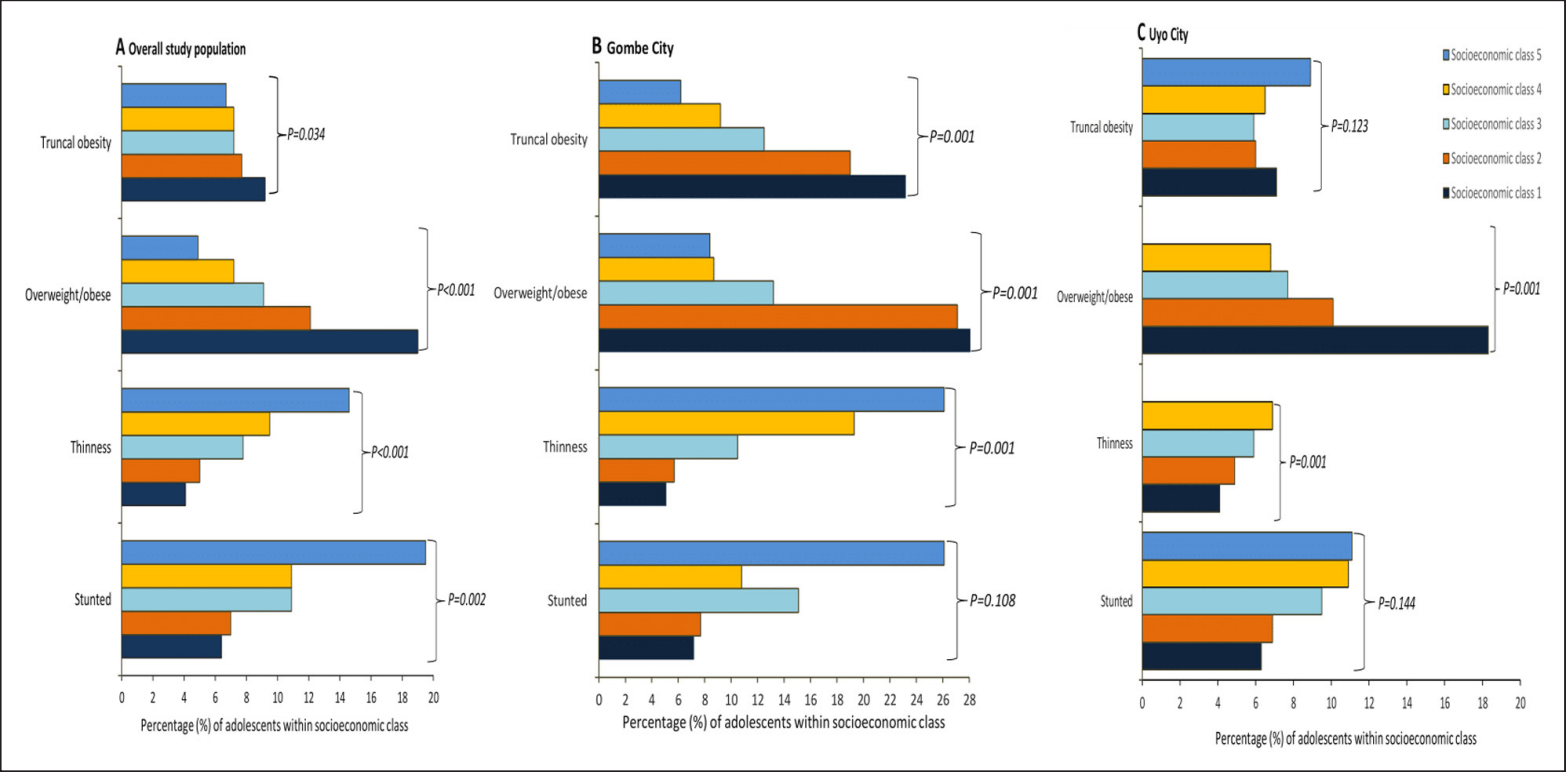
The prevalence rate of population-level malnutrition (truncal obesity, overweight/obesity, thinness and stunting) by socioeconomic status among adolescents in Gombe, Uyo and the overall study population. *Note*: Within-group prevalence rates are computed (i.e. the prevalence of over- or undernutrition within each socioeconomic class). Thinness = BAZ < –2SD, overweight/obesity = BAZ > 1SD, stunting = HAZ < –2SD, and truncal obesity = WHtR ≥ 0.5.

Overall, BAZ and WHtR overweight and obesity measures were comparatively higher in the north, in girls, and early adolescence (Table 2). Stunting (HAZ < –2SD) was comparatively more prevalent in the north (p = 0.035), in male adolescents (p = 0.000), and among participants in the middle adolescence age group (p = 0.000) (Table 2). At the population level, the burden of the DBM was higher in the north compared to the south and the differences were statistically significant (p = 0.000). Using the BAZ categorization, the population in northern Nigeria concurrently had a higher prevalence of thinness (under-nutrition) at 11.98% and a high prevalence of overweight/obesity (over-nutrition) at 16.08%. On the other hand, adolescents in southern Nigeria had thinness (under-nutrition) of 5.3% and overweight/obesity (over-nutrition) of 11.31% (Figure 1). Using measures of stunting (HAZ) and abdominal obesity (WHtR), the level of population-level DBM was also higher in the north compared to adolescents in southern Nigeria. In the north, 12.53% of adolescents were stunted/severely stunted while 13.9% had abdominal obesity. In southern Nigeria, stunted/severely stunted adolescents were 8.14% and abdominal obesity was 6.4% in the same population (Figure 1). Overall, the prevalence of truncal obesity and general overweight/obesity was significantly higher among adolescents in the wealthiest socioeconomic group (p = 0.034 and p < 0.001 respectively). In contrast, the prevalence of thinness and stunting was significantly higher among adolescent in the poorest socio-economic group with p < 0.001 and p = 0.002 respectively (Figure 2).

**Table 2:** Under-nutrition and over-nutrition measures by location, sex and age among 2,100 adolescents in Gombe and Uyo.

Various Obesity Measures	Location			Sex		Age Groups					

	Uyo, n (%)	Gombe, n (%)	P Value	Male, n (%)	Female, n (%)	P Value	Early, n (%)	Middle, n (%)	Late, n (%)	P Value	Total (%)
			
	N= 1733	N = 367		N = 927	N = 1,173		N = 684	N = 1,056	N = 360		N = 2,100

**BMI For Age**											
Severe Thinness	20 (1.15)	6 (1.63)		20 (2.16)	6 (0.51)		7 (1.02)	14 (1.33)	5 (1.39)		26 (1.24)
Thinness	72 (4.15)	38 (10.35)		74 (7.98)	36 (3.07)		28 (4.09)	55 (5.21)	27 (7.50)		110 (5.24)
Normal	1,445 (83.38)	264 (71.93)		756 (81.55)	953 (81.24)		518 (75.73)	895 (84.75)	296 (82.22)		1,709 (81.38)
Overweight	152 (8.77)	40 (10.90)		53 (5.72)	139 (11.85)		6 (12.57)	78 (7.39)	28 (7.78)		192 (9.14)
Obese	44 (2.54)	19 (5.18)	0.000	24 (2.59)	39 (3.32)	0.000	45 (6.58)	14 (1.33)	4 (1.11)	0.000	63 (3.00)
**Height for Age**											
Severely stunted	23 (1.33)	8 (2.18)		22 (2.37)	9 (0.77)		2 (0.29)	23 (2.17)	6 (1.67)		31 (1.48)
Stunted	118 (6.81)	38 (10.35)		113 (12.19)	43 (3.67)		31 (4.53)	98 (9.28)	27 (7.50)		156 (7.43)
Normal	1,585 (91.46)	321 (87.47)		790 (85.22)	1,116 (95.14)		644 (94.15)	935 (88.54)	327 (90.83)		1,906 (90.76)
Tall	7 (0.40)	0 (0.00)	0.035	2 (0.22)	5 (0.43)	0.000	7 (1.02)	0 (0.00)	0 (0.000)	0.000	7 (0.33)
**Waist to height ratio**											
Normal	1,622 (93.59)	316 (86.10)		892 (96.22)	1,046 (89.17)		617 (31.84)	992 (51.19)	329 (16.98)		1,938 (92.29)
Obese (truncal)	111 (6.41)	51 (13.90)	0.000	35 (3.78)	127 (10.83)	0.000	67 (41.36)	64 (39.51)	31 (19.14)	0.013	162 (7.71)

### Individual-level DBM

**Figure 3:**
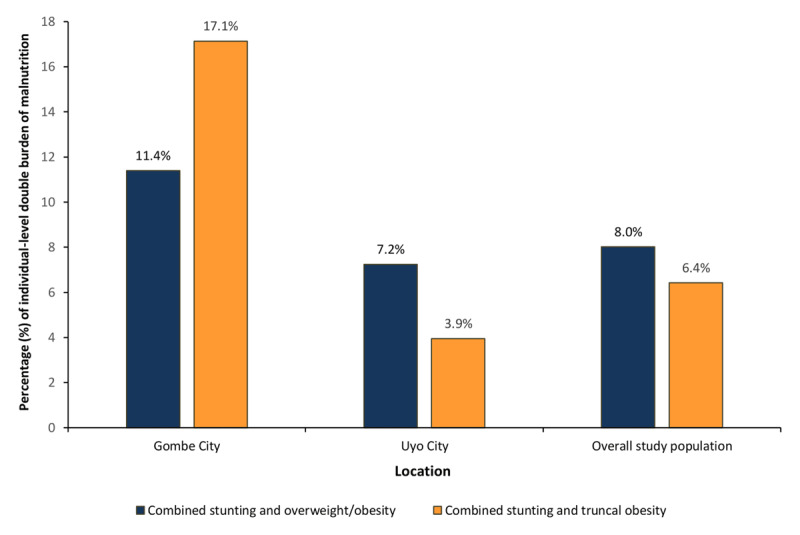
The prevalence rate of individual-level double burden malnutrition among adolescents in Gombe, Uyo and the overall study population. *Note*: This graph shows the proportion of adolescents who were either concurrently stunted (HAZ < –2SD) and overweight/obese (BAZ > 1SD) or Stunted (HAZ < –2SD) and had truncal obesity (WHtR ≥ 0.5).

There was a double burden of malnutrition at the individual level. Overall, among adolescents who were stunted, 6.42% had truncal obesity at the same time (when measured by WHtR). The burden rose to 8.02% when measured by BAZ (overweight/obesity) (Figure 3). This phenomenon was again more prevalent in the north with the coexistence of general overweight/obesity (BAZ) among adolescents who were stunted (HAZ) of 11.4% in Gombe compared to 7.24% at individual-level in Uyo. Furthermore, the difference in the coexistence of individual-level stunting (HAZ) and truncal overweight/obesity (WHtR) was more than four times higher in Gombe compared to Uyo (i.e. 17.14% vs 3.95% respectively) (Table 3).

**Table 3:** Comparing Individual-level coexistence of stunting and obesity/overweight among adolescents in Gombe and Uyo.

	HAZ category Overall		HAZ category Gombe		HAZ category Uyo		P Value

	*Stunted	Normal	*Stunted	Normal	*Stunted	Normal	

	N = 187 (%)	N = 1913 (%)	N = 35 (%)	N = 332 (%)	N = 152 (%)	N = 1,581 (%)	

**BMI**							
Thinness**	42 (22.46)	94 (4.91)	7 (20.00)	26 (7.83)	35 (23.03)	68 (4.30)	
Normal	130 (69.52)	1,579 (82.54)	24 (68.57)	278 (83.73)	106 (69.74)	1,301 (82.29)	
Overweight/Obesity	**15 (8.02)**	240 (12.55)	**4 (11.4)**	28 (8.43)	**11 (7.24)**	212 (13.41)	0.410
**WHtR**							
Normal	175 (93.58)	1,763 (92.16)	29 (82.86)	307 (92.47)	146 (96.05)	1,456 (92.09)	
Obese (truncal)	**12 (6.42)**	150 (7.84)	**6 (17.14)**	25 (7.53)	**6 (3.95)**	125 (7.91)	0.004

* Stunted included adolescents who were stunted (HAZ < –2SD) and those who were severely stunted (HAZ < –3SD). ** Thinness included adolescents who were thin (BAZ < –2SD) and those with severe thinness (BAZ < –3SD).

## Discussion

To the authors’ knowledge, this analysis represents the first comparative analysis of the status of the DBM targeted at the adolescent age group in two emerging cities in northern and southern Nigeria. Overall, both cities had a complex mix of stunting, thinness, overweight, general obesity, and truncal obesity prevalent among the adolescent population studied. Significantly more boys and those in middle/late adolescent were stunted and thin compared to girls and their counterparts in other age groups. More girls and those in early adolescence age group were overweight and had general obesity. Furthermore, truncal obesity was more prevalent among girls and those in the early and middle adolescence age group. These patterns, however, had a clear distinction between adolescents in northern compared to southern Nigeria with the study population in Gombe (northern Nigeria) having disproportionately higher levels of all forms of under-nutrition and over-nutrition.

This study found that population-level DBM (coexistence of thinness and overweight/obesity at the community level) exists in both settings studied, however, with a significantly higher burden in Gombe compared to Uyo in southern Nigeria. The findings in this study agree with data from elsewhere in sub-Saharan Africa which have reported that population-level DBM is prevalent both in rural and urban settings, however, with varying prevalences [15,16]. Additionally, the higher prevalence of DBM in Gombe (northern Nigeria) is supported by evidence from the 2018 National Nutrition and Health Survey (NNHS) conducted across Nigeria. The NNHS reported that levels of stunting, thinness, overweight and obesity were higher among U5 children in Gombe State (where Gombe city is located) compared to those in Akwa-Ibom State where Uyo is located [35]. The higher prevalence of the DBM found among Gombe adolescents, may therefore imply that the trends reported by the NNHS may not be limited to U5 children alone, but probably extends to adolescentce as well. This study suggests that despite being located in the same country, Gombe and Uyo may be at different stages of the nutrition and epidemiological transitions as evidenced by the differential levels of the DBM [36].

Underlying differences in poverty rates across Nigeria, reinforced by complex differences in climatic conditions, social, economic, and historical legacies could also partly explain the higher burden of population-level DBM in Gombe compared to Uyo. Despite several initiatives, rates of poverty, hunger, and chronic undernutrition (including stunting) have remained consistently high in northern compared to southern Nigeria [37]. This situation has recently been complicated by food insecurity occasioned by a decade-long armed conflict ravaging several parts of northern Nigeria including Gombe. In the context of high rates of poverty, and underlying chronic undernutrition [37], scarcity of local foods and recent urbanization may have contributed to a nutrition transition in Gombe, with increased consumption of cheap, highly processed energy-dense foods, which are low in nutritional quality [38]. The continued consumption of such foods fails to address the background chronic undernutrition (such as stunting) but instead contributes to an increased burden of overweight and obesity [38]. This prevailing situation could have accounted for the coexistence of the high burdens of under-nutrition and over-nutrition in Gombe compared with Uyo and may partly explain the higher rates of population-level DBM.

As with population-level DBM, the study found individual-level DBM (coexistence of under- and over-nutrition in the same individual) was more prevalent among adolescents in Gombe than those in Uyo. Due to the cross-sectional nature of this study, it may be difficult to show temporality related to the sequence of development of under- or over-nutrition among the adolescents studied. However, existing literature suggests that a possible explanation is that common background physiologic changes may be contributing simultaneously to under-nutrition (stunting) and excess adiposity (overweight/obesity) in the same individual at the same time [39]. The question remains if there is enough calorie in a child’s diet to gain excess adiposity, why then does the same child remain stunted and why is it different for children in Gombe and Uyo? Differences in diet composition and quality during childhood and varying genetic factors at the individual level might be responsible for the differences reported in this study [40,41].

Another plausible explanation for individual-level DBM is that early under-nutrition (stunting and underweight) may alter an individual’s physiologic makeup, increasing the likelihood of becoming overweight or obese in later life; the so-called ‘thrifty phenotype,’ which increases the efficiency of adiposity storage [39]. There is evidence to suggest that stunted children have a higher likelihood to accumulate truncal adiposity than their counterparts with normal height [42,43]. In fact, a longitudinal study in Brazil by Hoffman et al. [44] showed that children who were stunted in early childhood had a greater proportion of truncal adiposity and a higher increase in truncal fat mass when followed up till adolescence, compared to their normal height counterparts. In a background of significantly higher rates of chronic stunting in northern compared to southern Nigeria [35], the finding of more than four times higher rates of individual-level DBM (i.e. coexistence of stunting and truncal obesity measured by HAZ and WHtR respectively) in Gombe compared to Uyo supports the existing evidence in this regard.

Based on the evidence from this study, adolescence is an important period for detecting the presence of DBM at the individual and population levels. Childhood and adolescence are critical periods of physical and cognitive development, thus, ignoring the high burden of the two extremes of malnutrition reported in this study setting might pose some dire consequences at the individual, household, and population levels. Undernutrition (stunting and thinness) contributes to cognitive impairment with consequences such as delayed school entry and poor school performance which could reduce a child’s life chances [43]. On the other hand, overnutrition (overweight/obesity) also impacts negatively on a child’s potential contributions to society in later life because childhood obesity is associated with increased risk of noncommunicable diseases in adulthood [45]. The differences in the burden of the DBM in the two cities studied highlights the need for context-specific approaches in managing the emerging trends. At the individual level, interventions such as regular anthropometric monitoring in childhood to aid early detection, contextualized feeding programs targeting under- and over-nutrition, and encouraging physical activity among children and adolescents should be implemented. At the population-level, subnational governments in both settings should prioritize interventions to limit the sale of highly processed energy-dense foods which have been shown to have low nutritional value.

There are important limitations to this study which should be considered. First, due to the cross-sectional design of the study, causality and temporal relationships between under- and over-nutrition among adolescents at the individual-level DBM may be more complex than presented. However, there is existing evidence suggesting a temporal relationship between chronic undernutrition (stunting) in early childhood and the presence of truncal adiposity in later childhood [44]. Second, the generalizability of the finding to other settings within northern and southern Nigeria might be limited because Nigeria is a vast and complex society with diverse social, economic, climatic, and environmental factors which determine nutrition in the first instance. While generalizability might not be made, this study provides, for the first time, valuable evidence of the presence of individual-level and population-level DBM in two distinct settings in Nigeria. Finally, we did not study the diverse social and behavioural factors which could have accounted for the differences in the burden of DBM reported in this study population.

## Conclusion

This study provides evidence of the presence of DBM in two emerging cities in Nigeria. There was a complex mix of stunting, thinness, general overweight/obesity and truncal obesity prevalent among the adolescent population in northern and southern Nigeria. All forms of population-level and individual-level DBM were more prevalent in Gombe (northern Nigeria) compared to Uyo (southern Nigeria). These findings imply that the health system in Gombe may struggle to deal with this new and emerging reality of coexisting high burden of under-nutrition and over-nutrition and their complications. There is a window of opportunity in Uyo for the health system to adopt strategies aimed at halting a further rise in the rates of under- and over-nutrition among adolescents. There is need for context-specific and evidence-informed approaches to halting and reversing the emerging trends in both settings.

## Figures and Tables

**Table:** 

BAZ	BMI-for-Age Z-score
BMI	Body Mass Index
CI	Confidence Interval
DBM	Double Burden of Malnutrition
HAZ	Height-for-Age Z-score
LMICs	Low-and Middle-Income Countries
NCDs	Non-Communicable Diseases
NNHS	National Nutrition and Health Survey
SD	Standard Deviation
SES	Socioeconomic Status
sSA	sub-Saharan Africa
U5	Children Under Five Years old
WHO	World Health Organization
WHtR	Waist-to-Height-Ratio

## Data Availability

The datasets analysed during the current study are available from the corresponding author on reasonable request.
